# Synthesis of thienyl analogues of PCBM and investigation of morphology of mixtures in P3HT

**DOI:** 10.3762/bjoc.4.33

**Published:** 2008-09-29

**Authors:** Fukashi Matsumoto, Kazuyuki Moriwaki, Yuko Takao, Toshinobu Ohno

**Affiliations:** 1 Osaka Municipal Technical Research Institute, 1-6-50 Morinomiya, Joto-ku, Osaka 536-8553, Japan.

**Keywords:** fullerene, morphology, organic fullerenes, PCBM, photovoltaic cell

## Abstract

Novel [6,6]-phenyl-C61-butyric acid methyl ester (PCBM) analogues containing benzo[b]thiophene (**3a**, **3b**) and thieno[3,2-b]thiophene (**3c**, **3d**) were synthesized and characterized. The morphology of the thin films prepared from the mixtures of these methanofullerenes with regioregular poly(3-hexylthiophene) (P3HT) was investigated by AFM measurement and UV-Vis absorption spectroscopy. A solubility test of these methanofullerenes was performed by using dichloromethane as a solvent. es-TThCBM (**3d**) exhibited 1.4 times greater solubility in dichloromethane than PCBM.

## Introduction

**Figure 1:**
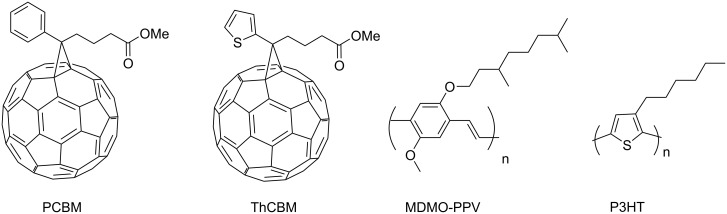
Molecular structures of PCBM, ThCBM, MDMO-PPV, and P3HT.

The demand for inexpensive, renewable energy sources continues to stimulate new approaches to the production of efficient, low-cost photovoltaic (PV) devices [1–3]. In particular, the use of polymers for the fabrication of solar cells would offer considerable advantages such as the low-cost production of large-area, flexible, and light solar cells [4–7]. Recently, bulk heterojunction solar cells consisting of a conjugated polymer (donor) and fullerene derivatives (acceptors) have inspired much scientific research owing to these reasons [8–9]. In this research, it has been demonstrated that the PV cells based on regio-regular poly(3-hexylthiophene) (P3HT, Figure 1) with [6,6]-phenyl-C61-butyric acid methyl ester (PCBM, Figure 1) are highly efficient and promising devices [10]. Many efforts have been devoted to improve device efficiency, for example, changing the solvent for the spin coating of the active layer [11–12], annealing the device to improve the crystallinity of the polymer [13–14], and controlling the growth rate of the organic film [15–16]. These studies reported that the morphology of the active layer is a critical factor that determines the device efficiency.

Investigations have been performed for exploring new donor materials for bulk heterojunction PV devices and the first drastic improvement in device efficiency was achieved by replacing the original donor, poly{[2-methoxy-5-(3,7-dimethyloctyloxy)-*p*-phenylene]vinylene} (MDMO-PPV, Figure 1), with a high-mobility conjugated polymer, P3HT [17–20]. However, sufficient investigations exploring new acceptor materials have not been performed.

In particular, there is no research focusing on how the structure of fullerene derivatives affects the morphology of the active layer of a PV device. However, it is essential to explore novel fullerene derivatives in order to achieve a significant improvement in the efficiency of PV cells. Recently, Hummelen et al. reported a device fabricated with a thienyl analogue (ThCBM, Figure 1) [21] for the purpose of improving the miscibility with P3HT. However, there is no obvious explanation related to the advantages of ThCBM over PCBM. In this study, we prepared novel PCBM analogues having various types of thienyl groups and investigated their structural effect on solubility and the morphology of their mixtures with P3HT.

## Results and Discussion

### Synthesis of thienyl analogues of PCBM

**Scheme 1:**
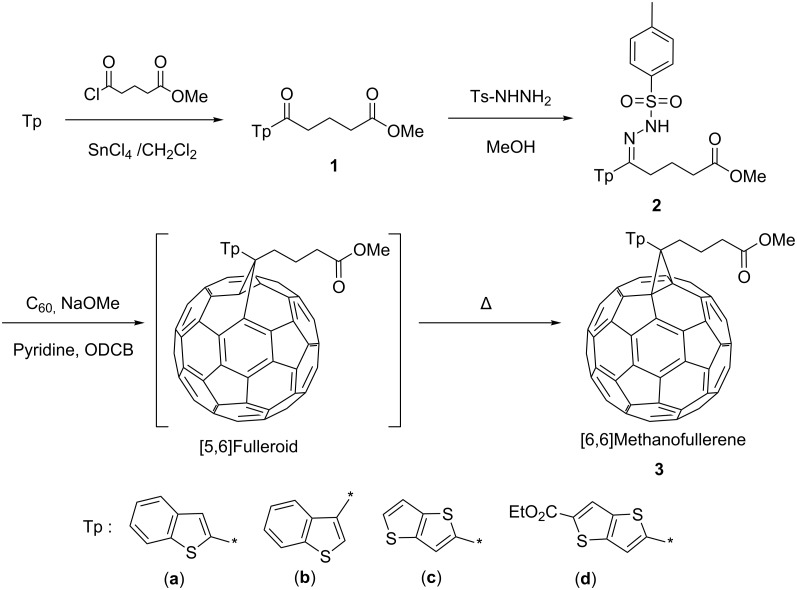
Synthesis of methanofullerenes (**3a**–**d**).

Thienyl-substituted methanofullerenes were synthesized by a general procedure [22], as shown in Scheme 1. First, benzo[b]thiophene and thieno[3,2-b]thiophenes were acylated by using SnCl_4_; the acylation was performed at low temperature as a safeguard against the high reactivity of the thienyl groups (yields: **1a**; 15%, **1b**; 46%, **1c**; 74%, **1d**; 33%). Glutaric acid monomethyl ester chloride was used as an acylation reagent in order to form the same structure as that of the butyric acid methyl ester contained in PCBM. The acylation of benzo[b]thiophene yielded 2- and 3-substituted compounds in a 1:3 ratio. These isomers were isolated with a recycling HPLC system using chloroform as an eluent and the compounds **1a** and **1b** were easily obtained. Then, the carbonyl group of **1** was converted to hydrazone by mixing **1** with *p*-toluenesulfonyl hydrazide. The compounds **2** were obtained as white to pale yellow solids in good yields (**2a**; 85%, **2b**; 90%, **2c**; 74%, **2d**; 70%). The methanofullerenes were prepared from *p*-tosylhydrazones (**2**) by a one-pot method. A slight excess of *p*-tosylhydrazone (1.2 equiv) was mixed and heated with C_60_ in the presence of sodium methoxide. The reaction was monitored by TLC and stopped within 3 to 4 h. As expected, the compound **3d** exhibited an extremely low R_f_ value because of the high polarity of the additional ethyl ester group. During the reaction, mono, di, or highly substituted methanofullerenes were obtained simultaneously; therefore, the monosubstituted methanofullerenes were separated from the mixtures by column chromatography. The products were further purified by reprecipitation from methanol.

In general, the resulting products of the above synthesis process contain the isomers of [5,6]fulleroid (methanoannulene-type bonding) and [6,6]methanofullerene (cyclopropane bonding) [23]. These isomers are clearly distinguishable by the ^13^C NMR chemical shifts of bridging units. It is well known that [5,6]fulleroid has low solubility and is thermodynamically unstable; hence, it cannot be used in a photovoltaic device. However, except for **2b**, the thienyl-substituted hydrazones **2a**, **2c**, and **2d** yielded only [6,6]methanofullerenes. The hydrazone **2b** yielded a mixture of [5,6] and [6,6] isomers, probably because of the steric hindrance of benzo[b]thiophen-3-yl or the difference in the electron-donating property between benzo[b]thiophene-3-yl and 2-yl. Therefore, the product **3b** needed heat treatment to convert to [6,6]methanofullerene. In short, thienyl analogues have an advantage over PCBM that they do not need the isomerization step at a temperature as high as 180 °C. The structures of the obtained methanofullerenes (**3**) were determined by ^1^H and ^13^C NMR, IR, and FD-MS spectra. The reaction conditions and product yields are summarized in Table 1.

**Table 1:** Reaction conditions and yields of methanofullerenes.

Product	**2**/C_60_	Temp (°C)	Time (h)	Condition^a^	Yield (%)

2-BThCBM (**3a**)	1.2	100	4	–	39
3-BThCBM (**3b**)	1.2	100	4	180 °C, 12 h	32
TThCBM (**3c**)	1.2	100	4	–	33
es-TThCBM (**3d**)	1.2	100	3.5	–	42

^a^Thermal conversion from [5,6]fulleroid to [6,6]methanofullerene in ODCB.

### Solubility of methanofullerenes

Good solubility of fullerenes is important for fabricating a photovoltaic device with good morphology [24]. In fact, PCBM is one of the most soluble fullerene derivatives and this is the main reason why it is used for the fabrication of PV cells. Chlorobenzene is normally used as a solvent of P3HT and fullerenes to fabricate a bulk hetero layer by spin coating; however, chlorobenzene almost completely dissolves methanofullerenes and hence cannot be used to determine and compare the solubility of different methanofullerenes at a small scale; therefore, in this study, dichloromethane was used as a solvent in the solubility test. As shown in Table 2, the solubilities of the obtained methanofullerenes (**3**) and ThCBM were significantly different despite little difference in their structures. Surprisingly, es-TThCBM (**3d**) exhibited the best solubility of 1.4 times that of PCBM; in contrast, TThCBM (**3c**) exhibited the lowest solubility. This result plainly indicates that an ester group is a key structure to yield a good solubility. It is very interesting that the solubilities of regioisomers of 2-BThCBM (**3a**) and 3-BThCBM (**3b**) were extremely different; however, the reasons related to the difference of structures are unknown. ThCBM exhibited a lower solubility than those of PCBM and 2-BThCBM (**3a**).

**Table 2:** Solubility of methanofullerenes^a^.

	PCBM	ThCBM	2-BThCBM (**3a**)	3-BThCBM (**3b**)	TThCBM (**3c**)	es-TThCBM (**3d**)

mmol/L	17.6	10.4	13.2	3.51	3.05	23.8
g/L	16	9.56	13.1	3.49	2.96	24.9

^a^maximum solubility in dichloromethane.

It is important that the solubility of es-TThCBM (**3d**) exceeds that of PCBM, which is promising to obtain better and thick active layers for PV cells.

### Investigation of morphology

Bulk hetero films of these novel methanofullerenes were spin coated on glass substrates and were characterized by using AFM and UV-Vis absorption spectroscopy in order to study and compare their morphological features. 3-BThCBM (**3b**) and TThCBM (**3c**) were excluded because their high-concentration solutions could not be prepared in the solvents used in this study. The bulk hetero films were prepared as follows. Regioregular P3HT and methanofullerene (either **3a** or **3d**) were dissolved in chlorobenzene at a 1:0.8 wt ratio, followed by filtration. Then, the solution was directly spin coated on a freshly rinsed glass plate and dried for 24 h at room temperature under vacuum.

**Figure 2:**
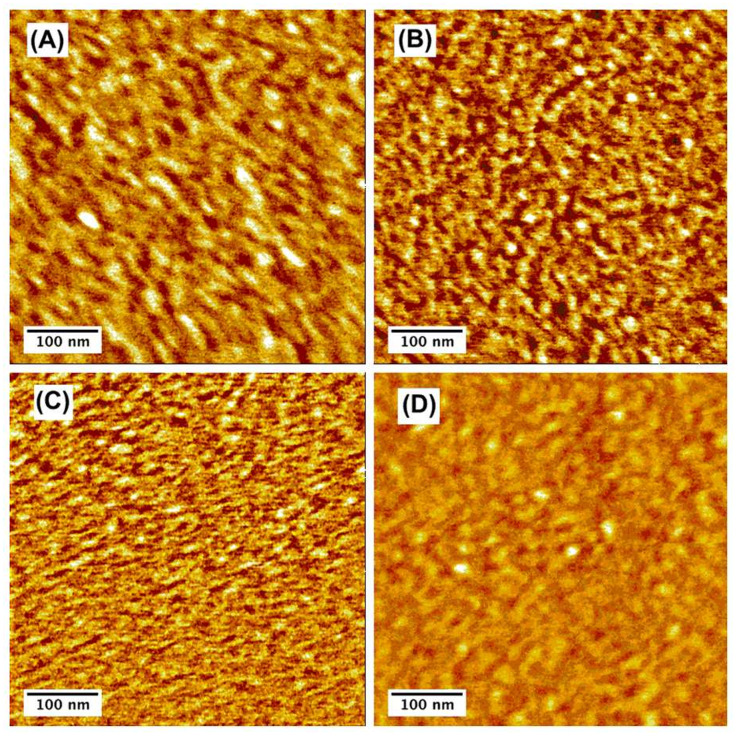
AFM phase images of P3HT/fullerene blend films containing (A) PCBM, (B) ThCBM, (C) 2-BThCBM (**3a**), and (D) es-TThCBM (**3d**) for a 500 × 500-nm^2^ area.

Tapping-mode AFM measurements were performed on a film area of 500 × 500 nm. All films had a very flat surface and almost the same roughness (r.m.s. value of 0.2–0.24 nm). However in the phase image (Figure 2), (A) PCBM and (B) ThCBM appear to have coarse features as compared with (C) 2-BThCBM (**3a**) and (D) es-TThCBM (**3d**). These chainlike features (bright area) are assigned to the domains of P3HT crystallites. The area between these features consists of either fullerene clusters or a P3HT/fullerene mixture [25]. The domain sizes in the images of PCBM and ThCBM are slightly larger (4–5 nm) than those of 2-BThCBM (**3a**) and es-TThCBM (**3d**). The separation distances of these features in PCBM and ThCBM are also longer by 5 to 10 nm than those in 2-BThCBM (**3a**) and es-TThCBM (**3d**). This result indicates that PCBM and ThCBM have large crystallites of P3HT, while 2-BThCBM (**3a**) and es-TThCBM (**3d**) mainly consist of amorphous domains of a P3HT/fullerene mixture.

**Figure 3:**
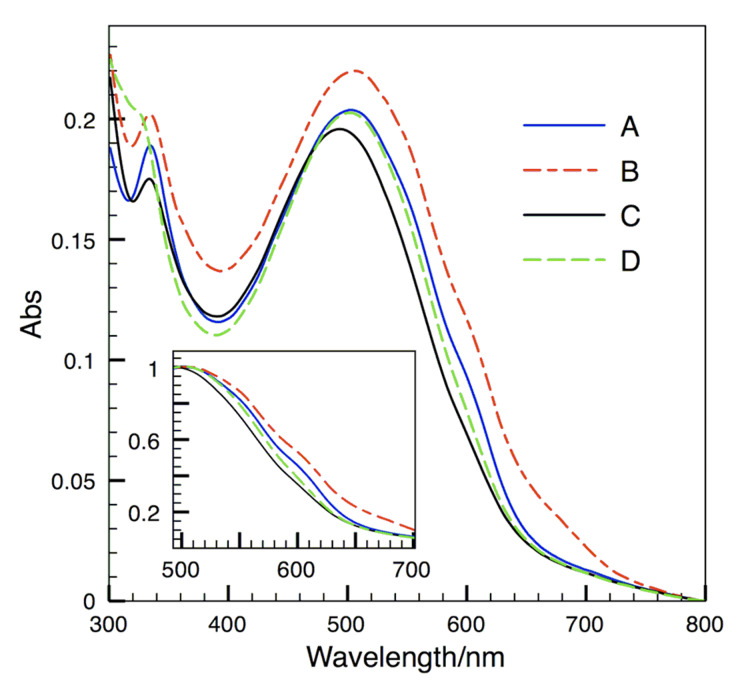
UV-Vis absorption spectra of P3HT/fullerene blend films for (A) PCBM, (B) ThCBM, (C) 2-BThCBM (**3a**), and (D) es-TThCBM (**3d**). Inset: normalized spectra at 500 nm.

This distinction was also observed in UV-Vis absorption spectra (Figure 3). (A) PCBM and (B) ThCBM exhibited absorption shoulders around 550 and 600 nm, while (C) 2-BThCBM (**3a**) and (D) es-TThCBM (**3d**) showed smooth slopes without such shoulders. As is generally known, the peak at 500 nm is derived from P3HT, almost independent from methanofullerene. Thus, the difference in the shapes of the peaks should be attributed to the variation of the solid state of P3HT [26]. When P3HT is in a crystal state, additional peaks are observed at a higher wavelength because of the π-π interaction between its polymer chains. Therefore, the P3HT contained in the films of PCBM and ThCBM is in a more crystalline state than that contained in the films of 2-BThCBM (**3a**) and es-TThCBM (**3d**), which is almost amorphous.

Assuming that the solubility trend of the methanofullerenes is the same in chlorobenzene and dichloromethane, PCBM and es-TThCBM (**3d**) seem to disperse very well in the mixture and result in more fine morphology. However, the variation of the film morphology had no correlation with the solubility trend of methanofullerenes and was therefore attributed to the structures of the methanofullerenes: 2-BThCBM (**3a**) and es-TThCBM (**3d**) are slightly larger than PCBM and ThCBM. The methanofullerenes (**3a**, **3d**) seem to interfere the aggregation of P3HT, which leads to a more amorphous phase. Comparing PCBM and ThCBM, there is no evident difference in their morphology. The improvement in miscibility between P3HT and PCBM by replacing the phenyl group of PCBM by a thienyl group was not clearly confirmed at this stage.

## Conclusion

Novel PCBM analogues containing thienyl groups were synthesized. The morphology of the bulk hetero films of the obtained methanofullerenes was different from that of PCBM. To some extent, the aggregation of P3HT was restricted by the large volume of 2-BThCBM (**3a**) and es-TThCBM (**3d**). Among the obtained methanofullerenes, es-TThCBM (**3d**) exhibited the best solubility. It is a rare methanofullerene that has greater solubility than PCBM. The improvement in solubility by just adding an ester group is very informative and promising for further development of methanofullerenes. Bulk heterojunction solar cells using newly synthesized methanofullerenes are being fabricated and evaluated.

## Supporting Information

File 1 Experimental part. Experimental procedures and data for all new compounds
