# Supplementary material for: Synthesis of thienyl analogues of PCBM and investigation of morphology of mixtures in P3HT
Source: Beilstein J Org Chem. 2008 Sep 29;4:33. doi: 10.3762/bjoc.4.33 (PMC2568877; doi:10.3762/bjoc.4.33)
Supplement: File 1 — Experimental part. Experimental procedures and data for all new compounds [file Beilstein_J_Org_Chem-04-33-s001.doc]

Synthesis of thienyl analogues of PCBM and investigation of morphology of mixtures in P3HT

Fukashi Matsumoto*, Kazuyuki Moriwaki, Yuko Takao and Toshinobu Ohno*

Address: Osaka Municipal Technical Research Institute, 1-6-50 Morinomiya, Joto-ku, Osaka 536-8553, Japan.

Email: Fukashi Matsumoto* - matumoto@omtri.city.osaka.jp; Toshinobu Ohno* - ohno@omtri.city.osaka.jp

*Corresponding author

**Experimental part**

Materials

Compounds thieno[3,2-b]thiophene [1], ethyl thieno[3,2-b]thiophene-2-carboxylate [1], 1-(3-(methoxycarbonyl)propyl)-1-phenyl-[6,6]methanofullerene (PCBM) [2] and 1-(3-(methoxycarbonyl)propyl)-1-thienyl-[6,6]methanofullerene (ThCBM) [3] were prepared according to the literature. Benzo[b]thiophene and other reagents were commercially available and used as received. All reactions involving air-sensitive reagents were performed under an atmosphere of dry nitrogen.

Instrumentation

^1^H NMR and ^13^C NMR spectra were recorded using a JEOL AL-300 (300 MHz) in deuterated solvents with tetramethylsilane as an internal reference. IR spectra measurements were carried out using a Jasco FT/IR-4100. UV-Vis absorption spectra were recorded using a Shimadzu UV-3100A. Gas-chromatography-mass spectrometry (GC-MS) measurements were performed using a Varian 3400. FD-Mass spectra were obtained on a JEOL JMS-DX-303HF. Isomers were isolated using a JAI LC-908 Recycling Preparative HPLC. Elemental analyses were performed on a CE instruments EA-1110. AFM images were taken with a Digital Instruments Nanoscope IIIa at scan rate of 1.0 Hz in the taping mode.

Solubility determination

Solubility data were obtained as follows. *Ca*. 1.5 mL of CH_2_Cl_2_ was added to an excess of methanofullerene solids. The mixture was sonicated at room temperature for 15 min and centrifuged for 15 min. The solution phase was passed through a 0.45 μm syringe filter. The 1.0 mL sample of this saturated solution was diluted into 50 mL with CH_2_Cl_2_. Absorbance measurements were carried out with a Shimazu UV-3100A. The solubilities of the methanofullerenes were calculated from the absorbance value at 430 nm by measuring the absorbance of standard solution of the methanofullerenes (0.1 g/L) in CH_2_Cl_2_.

**Methyl 5-(benzo[*b*]thiophen-2-yl)-5-oxopentanoate (1a).** To a mixture of benzo[b]thiophene (2.01 g, 15.0 mmol) and glutaric acid monomethyl ester chloride (2.96 g, 18.0 mmol) in 50 mL of anhydrous CH_2_Cl_2_, 1.0 mol/L solution of tin(IV) chloride (8.60 g, 33.0 mmol) in CH_2_Cl_2_ was added dropwise at 0 ˚C. The mixture was warmed to ambient temperature and stirred for 16 h, and quenched with 1.0 M hydrochloric acid. The organic material was extracted with dichloromethane and dried over Na_2_SO_4_. The crude product was purified on a silica gel column using an ethyl acetate/hexane mixture (1:3 by volume) as an eluent, and recrystallized from methanol. Separating the structural isomers of **1a** and **1b** by using a recycled HPLC gave 0.602 g of pure compound **1a** in 15% yield. ^1^H NMR (CDCl_3_): δ 7.97 (s, 1H, Ar), 7.90-7.85 (m, 2H, Ar), 7.48-7.37 (m, 2H, Ar), 3.69 (s, 3H, OCH_3_), 3.10 (t, 2H, *J* = 7.2 Hz, CH_2_), 2.47 (t, 2H, *J* = 7.2 Hz, CH_2_), 2.16-2.07 (m, 2H, CH_2_). ^13^C NMR (CDCl_3_): δ 193.83 (C=O), 173.52 (C=O), 143.51, 142.44, 139.11, 129.00 (ArH), 127.37 (ArH), 125.90 (ArH), 124.97 (ArH), 122.96 (ArH), 51.57 (OCH_3_), 38.05 (CH_2_), 32.99 (CH_2_), 19.65 (CH_2_). IR (KBr): 3088, 3059, 2969, 2950, 2900, 1726 (C=O; s), 1660 (C=O; s), 1518, 1455, 1329, 1252, 1174, 1062, 1018, 844, 754 cm^-1^. Anal. Calcd for C_14_H_14_O_3_S: C, 64.10; H, 5.38; S, 12.22. Found: C, 64.23; H, 5.17. GC/MS: *m/e* 263 (M^+1^).

**Methyl 5-(benzo[*b*]thiophen-3-yl)-5-oxopentanoate (1b).** White product (1.80 g) was obtained in 46% yield by isolation from the crude isomers prepared above. ^1^H NMR (CDCl_3_): δ 8.76 (d, 1H, *J* = 8.1 Hz, Ar), 8.33 (s, 1H, Ar), 7.86 (d, 1H, *J* = 7.9 Hz, Ar), 7.45 (dd, 2H, *J* = 15.5 Hz, 7.2 Hz, Ar), 3.69 (s, 3H, OCH_3_), 3.08 (t, 2H, *J* = 7.2 Hz, CH_2_), 2.48 (t, 2H, *J* = 7.1 Hz, CH_2_), 2.17-2.09 (m, 2H, CH_2_). ^13^C NMR (CDCl_3_): δ 194.67 (C=O), 173.69 (C=O), 139.82, 136.72 (ArH), 136.62, 135.05, 125.80 (ArH), 125.67 (ArH), 125.40 (ArH), 122.20 (ArH), 51.57 (OCH_3_), 39.01 (CH_2_), 33.08 (CH_2_), 19.73 (CH_2_). IR (KBr): 3109, 2953, 2897, 1739 (C=O; s), 1668 (C=O; s), 1495, 1456, 1434, 1418, 1381, 1334, 1265, 1194, 1160, 1059, 989, 925, 869, 772, 736 cm^-1^. Anal. Calcd for C_14_H_14_O_3_S: C, 64.10; H, 5.38; S, 12.22. Found: C, 64.22; H, 5.53. GC/MS: *m/e* 263 (M^+1^).

**Methyl 5-(thieno[3,2-*b*]thiophen-2-yl)-5-oxopentanoate (1c)**. The compound was prepared by almost the same procedure as **1a**. From 1.63 g (11.6 mmol) of thieno[3,2-b]thiophene and 2.30 g (13.9 mmol) of glutaric acid monomethyl ester chloride, 2.32 g of **1c** was prepared in 74% yield, purified by recrystallization from chloroform/hexane mixture. ^1^H NMR (CDCl_3_): δ 7.93 (s, 1H, Ar), 7.62 (d, 1H, *J* = 5.1 Hz, Ar), 7.29 (d, 1H, *J* = 5.3 Hz, Ar), 3.69 (s, 3H, OCH_3_), 3.03 (t, 2H, *J* = 7.2 Hz, CH_2_), 2.46 (t, 2H, *J* = 7.1 Hz, CH_2_), 2.15-2.06 (m, 2H, CH_2_). ^13^C NMR (CDCl_3_): δ 193.00 (C=O), 173.54 (C=O), 145.55, 144.84, 139.13, 132.55 (ArH), 124.44 (ArH), 120.01 (ArH), 51.57 (OCH_3_), 37.79 (CH_2_), 33.01 (CH_2_), 19.85 (CH_2_). IR (KBr): 3091, 2955, 2895, 1709 (C=O; s), 1652 (C=O; s), 1498, 1413, 1263, 1202, 1163, 1070, 1013, 857, 745 cm^-1^. Anal. Calcd for C_12_H_12_O_3_S_2_: C, 53.71; H, 4.51; S, 23.90. Found: C, 53.63; H, 4.33. GC/MS: *m/e* 269 (M^+1^).

**Ethyl 5-(5-methoxy-5-oxopentanoyl)thieno[3,2-b]thiophene-2-carboxylate (1d).** From thieno[3,2-b]thiophene-2-carboxylate (2.65 g, 12.5 mmol) and glutaric acid monomethyl ester chloride (2.47 g, 15.0 mmol), compound **1d** was obtained as a white powder (1.40 g, 33% yield). ^1^H NMR (CDCl_3_): δ 7.98 (s, 1H, Ar), 7.92 (s, 1H, Ar), 4.40 (q, 2H, *J* = 7.2 Hz, CH_3_C*H_2_*O), 3.69 (s, 3H, OCH_3_), 3.05 (t, 2H, *J* = 7.2 Hz, COCH_2_), 2.46 (t, 2H, *J* = 7.1 Hz, CH_3_OCOC*H_2_*), 2.12-2.09 (m, 2H, CH_2_C*H_2_*CH_2_), 1.41 (t, 3H, *J* = 7.1 Hz, OCH_2_C*H_3_*). ^13^C NMR (CDCl_3_): δ 192.84 (C=O), 173.47 (C=O), 161.93 (C=O), 148.64, 143.39, 142.92, 139.69, 125.69 (ArH), 124.31 (ArH), 61.81 (OCH_2_), 51.62 (OCH_3_), 37.98 (CH_2_), 32.91 (CH_2_), 19.63 (CH_2_), 14.28 (OCH_2_*C*H_3_). IR (KBr): 3093, 2952, 2905, 1728 (C=O; s), 1712 (C=O; s), 1653 (C=O; s), 1481, 1287, 1242, 1183, 1083, 1019, 869, 746 cm^-1^. Anal. Calcd for C_15_H_16_O_5_S_2_: C, 52.92; H, 4.74; S, 18.84. Found: C, 53.32; H, 4.47. GC/MS: *m/e* 341 (M^+1^).

**Methyl 5-(benzo[b]thiophen-2-yl)-5-(2-tosylhydrazono)pentanoate (2a).** A mixture of 400 mg (1.52 mmol) of **1a** and 340 mg (1.82 mmol) of *p*-toluenesulfonyl hydrazide was dissolved in methanol (3 mL) and refluxed under air for 6 h. After cooling to room temperature, the crude product was collected by filtration. Recrystallization from methanol gave white crystals of **2a** in 85% yield (556 mg). ^1^H NMR (CDCl_3_): δ 9.16 (s, 1H, NH), 7.95 (d, 2H, *J* = 8.3 Hz, Ar), 7.80-7.77 (m, 1H, Ar), 7.70-7.68 (m, 1H, Ar), 7.38 (s, 1H, Ar), 7.31 (d, 2H, *J* = 8.3 Hz, Ar), 7.36-7.27 (m, 2H, Ar), 3.80 (s, 3H, OCH_3_), 2.68 (t, 2H, *J* = 8.0 Hz, CH_2_), 2.40 (s, 3H, ArCH_3_), 2.34 (t, 2H, *J* = 5.1 Hz, CH_2_), 1.80-1.77 (m, 2H, CH_2_). ^13^C NMR (CDCl_3_): δ 174.72 (C=O), 149.81 (C=N), 143.87, 142.52, 140.60, 139.50, 135.71, 129.47 (ArH), 128.11 (ArH), 125.66 (ArH), 124.38 (ArH), 124.00 (ArH), 123.10 (ArH), 122.28 (ArH), 52.42 (OCH_3_), 31.93 (CH_2_), 26.23 (CH_2_), 21.58 (ArCH_3_), 21.17 (CH_2_). IR (KBr): 3186, 2947, 1726 (C=O; s), 1597, 1407, 1336, 1245, 1171, 1070, 926, 861, 813, 670, 551 cm^-1^. Anal. Calcd for C_21_H_22_N_2_O_4_S_2_: C, 58.58; H, 5.15; N, 6.51; S, 14.90. Found: C, 58.46; H, 5.31; N, 6.31.

**Methyl 5-(benzo[b]thiophen-3-yl)-5-(2-tosylhydrazono)pentanoate (2b).** Reaction of 1.00 g (3.81 mmol) of **1b** and 0.85 g (4.57 mmol) of *p*-toluenesulfonyl hydrazide following the procedure described above gave **2b** as white crystals (1.48 g, 90%). ^1^H NMR (CDCl_3_): δ 9.22 (s, 1H, NH), 8.62-8.59 (m, 1H, Ar), 7.96 (d, 2H, *J* = 8.3 Hz, Ar), 7.81-7.78 (m, 1H, Ar), 7.60 (s, 1H, Ar), 7.38-7.37 (m, 2H, Ar), 7.29 (d, 2H, *J* = 8.3 Hz, Ar), 3.83 (s, 3H, OCH_3_), 2.71 (t, 2H, *J* = 8.2 Hz, CH_2_), 2.39 (s, 3H, ArCH_3_), 2.36 (t, 2H, *J* = 5.1 Hz, CH_2_), 1.83-1.76 (m, 2H, CH_2_). ^13^C NMR (CDCl_3_): δ 174.81 (C=O), 151.28 (C=N), 143.86, 140.34, 136.42, 136.08, 132.82, 129.59 (ArH), 128.09 (ArH), 128.04 (ArH), 127.77 (ArH), 126.62 (ArH), 124.96 (ArH), 122.22 (ArH), 52.40 (OCH_3_), 32.12 (CH_2_), 27.68 (CH_2_), 21.57 (ArCH_3_), 21.34 (CH_2_). IR (KBr): 3176, 2943, 1710 (C=O; s), 1595, 1433, 1340, 1221, 1162, 1034, 929, 881, 817, 764, 660 cm^-1^. Anal. Calcd for C_21_H_22_N_2_O_4_S_2_: C, 58.58; H, 5.15; N, 6.51; S, 14.90. Found: C, 58.44; H, 5.37; N, 6.49.

**Methyl 5-(thieno[3,2-b]thiophen-2-yl)-5-(2-tosylhydrazono)pentanoate (2c).** From 0.91 g (3.40 mmol) of **1c** and 0.76 g (4.08 mmol) of *p*-toluenesulfonyl hydrazide and following the procedure described above, **2c** was obtained in 74% yield (1.11 g). ^1^H NMR (CDCl_3_): δ 9.05 (s, 1H, NH), 7.92 (d, 2H, *J* = 8.1 Hz, Ar), 7.40 (d, 1H, *J* = 5.1 Hz, Ar), 7.36 (s, 1H, Ar), 7.30 (d, 2H, *J* = 8.1 Hz, Ar), 7.19 (d, 1H, *J* = 5.1 Hz, Ar), 3.77 (s, 3H, OCH_3_), 2.63 (t, 2H, *J* = 8.0 Hz, CH_2_), 2.39 (s, 3H, ArCH_3_), 2.33 (t, 2H, *J* = 6.1 Hz, CH_2_), 1.78-1.73 (m, 2H, CH_2_). ^13^C NMR (CDCl_3_): δ 174.56 (C=O), 150.42 (C=N), 144.14, 143.83, 140.90, 138.62, 135.63, 129.43 (ArH), 128.94 (ArH), 128.05 (ArH), 119.73 (ArH), 118.78 (ArH), 52.27 (OCH_3_), 31.95 (CH_2_), 26.37 (CH_2_), 21.54 (ArCH_3_), 21.20 (CH_2_). IR (KBr): 3143, 3096, 3079, 2957, 2928, 1709 (C=O; s), 1654, 1594, 1513, 1418, 1339, 1225, 1167, 1054, 819, 671 cm^-1^. Anal. Calcd for C_19_H_20_N_2_O_4_S_3_: C, 52.27; H, 4.62; N, 6.42; S, 22.03. Found: C, 52.59; H, 4.79; N, 6.14.

**Ethyl 5-(5-methoxy-5-oxo-1-(2-tosylhydrazono)pentyl)thieno[3,2-b]thiophene-2-carboxylate (2d).** From 1.02 g (3.00 mmol) of **1d** and 0.67 g (3.60 mmol) of *p*-toluenesulfonyl hydrazide and following the procedure described above, **2d** was obtained in 70% yield (1.07 g). ^1^H NMR (CDCl_3_): δ 9.29 (s, 1H, NH), 7.91 (d, 2H, *J* = 8.3 Hz, Ar), 7.88 (s, 1H, Ar), 7.36 (s, 1H, Ar), 7.31 (d, 2H, *J* = 8.3 Hz, Ar), 4.37 (q, 2H, *J* = 7.1 Hz, CH_3_C*H_2_*O), 3.77 (s, 3H, OCH_3_), 2.65 (t, 2H, *J* = 8.0 Hz, NCCH_2_), 2.40 (s, 3H, ArCH_3_), 2.35 (t, 2H, *J* = 6.1 Hz, CH_3_OCOC*H_2_*), 1.79-1.74 (m, 2H, CH_2_C*H_2_*CH_2_), 1.39 (t, 3H, *J* = 7.1 Hz, OCH_2_C*H_3_*). ^13^C NMR (CDCl_3_): δ 174.63 (C=O), 162.33 (C=O), 149.54 (C=N), 148.17, 143.98, 143.17, 139.93, 136.27, 135.55, 129.48 (ArH), 128.00 (ArH), 125.61 (ArH), 118.69 (ArH), 61.45 (OCH_2_), 52.31 (OCH_3_), 31.89 (CH_2_), 26.35 (CH_2_), 21.54 (ArCH_3_), 21.04 (CH_2_), 14.27 (OCH_2_*C*H_3_). IR (KBr): 3225, 3100, 2949, 1731 (C=O; s), 1696 (C=O; s), 1595, 1487, 1370, 1279, 1074, 849, 715, 666 cm^-1^. Anal. Calcd for C_22_H_24_N_2_O_6_S_3_: C, 51.95; H, 4.76; N, 5.51; S, 18.91. Found: C, 51.89; H, 5.07; N, 5.53.

**1-(3-(Methoxycarbonyl)propyl)-1-(benzo[b]thiophen-2-yl)-[6,6]methanofullerene (2-BThCBM, 3a).** A mixture of **2a** (200 mg, 0.47 mmol) and NaOMe (29.3 mg, 0.54 mmol) in 6 mL of dry pyridine was stirred for 15 min at ambient temperature. Then a solution of C_60_ (279 mg, 0.39 mmol) in dry 1,2-dichlorobenzene (18 mL) was added, the reaction mixture was stirred at 100 ˚C for 4 h. The solution was concentrated and poured on a silica gel/toluene column. The obtained product was dissolved in a small amount of chloroform and poured into methanol. The resulting suspension was centrifuged, the supernatant was decanted, and the residue was dried *in vacuo* at 80 ˚C for 48 h. 2-BThCBM (**3a**) was obtained as brown powder in 39% yield (153 mg). ^1^H NMR (CDCl_3_): δ 7.92-7.87 (m, 2H, Ar), 7.72 (s, 1H, Ar), 7.47-7.39 (m, 2H, Ar), 3.67 (s, 3H, OCH_3_), 3.02 (t, 2H, *J* = 8.0 Hz, CCH_2_), 2.58 (t, 2H, *J* = 7.4 Hz, COCH_2_), 2.33-2.23 (m, 2H, CH_2_). ^13^C NMR (CDCl_3_): δ 173.37 (C=O), 147.99, 147.22, 145.66, 145.25, 145.17, 145.08, 144.84, 144.74, 144.68, 144.63, 144.56, 144.21, 143.82, 143.79, 143.13, 143.09, 143.03, 142.97, 142.95, 142.22, 142.15, 141.02, 140.80, 140.15, 139.74, 138.73, 138.29, 138.24, 129.14 (ArH), 125.04 (ArH), 124.67 (ArH), 124.13 (ArH), 122.45 (ArH), 79.58 (bridgehead C), 51.67 (OCH_3_), 46.18 (bridge C), 33.67 (CH_2_), 33.45 (CH_2_), 22.55 (CH_2_). IR (KBr): 2941, 1736 (C=O; s), 1458, 1431, 1248, 1187, 1061, 833, 743, 723, 670 cm^-1^. Anal. Calcd for C_74_H_14_O_2_S: C, 91.92; H, 1.46; S, 3.32. Found: C, 91.75; H, 1.84. FD-MS: m/z = 966.28.

**1-(3-(Methoxycarbonyl)propyl)-1-(benzo[b]thiophen-3-yl)-[6,6]methanofullerene (3-BThCBM, 3b).** To a stirred mixture of **2b** (143 mg, 0.33 mmol) and NaOMe (21.0 mg, 0.39 mmol) in dry pyridine (4 mL), a solution of C_60_ (200 mg, 0.28 mmol) in dry 1,2-dichlorobenzene (12 mL) was added. The mixture was stirred at 100 ˚C for 4 h. The crude product was purified with a silica gel/toluene column. The obtained product was dissolved in 10 mL of dry 1,2-dichlorobenzene and heated at 180 ˚C under nitrogen atmosphere for 12 h. Then the compound **3b** was collected with the same manner as **3a** (88 mg, 32%). ^1^H NMR (CDCl_3_): δ 8.43 (d, 1H, *J* = 7.9 Hz, Ar), 8.00 (d, 1H, *J* = 7.9 Hz, Ar), 7.87 (s, 1H, Ar), 7.51 (td, 2H, *J* = 14.6, 7.3 Hz, Ar), 3.64 (s, 3H, OCH_3_), 3.18-3.11 (m, 1H, CCH_2_), 2.97-2.91 (m, 1H, CCH_2_), 2.53 (t, 2H, *J* = 7.7 Hz, COCH_2_), 2.30-2.17 (m, 2H, CH_2_). ^13^C NMR (CDCl_3_): δ 173.39 (C=O), 148.59, 148.41, 147.45, 145.87, 145.69, 145.27, 145.19, 145.12, 144.78, 144.56, 144.24, 144.02, 143.88, 143.80, 143.72, 143.13, 143.01, 142.31, 142.17, 142.06, 141.12, 140.52, 138.71, 138.41, 138.05, 137.83, 136.95, 130.78 (ArH), 129.99, 124.99 (ArH), 124.88 (ArH), 123.37 (ArH), 122.43 (ArH), 79.79 (bridgehead C), 51.64 (OCH_3_), 45.75 (bridge C), 33.73 (CH_2_), 30.86 (CH_2_), 23.03 (CH_2_). IR (KBr): 2941, 1736 (C=O; s), 1457, 1429, 1340, 1259, 1186, 1094, 1053, 1020, 803, 762, 733, 700, 572, 526 (s), 419 cm^-1^. Anal. Calcd for C_74_H_14_O_2_S: C, 91.92; H, 1.46; S, 3.32. Found: C, 90.17; H, 1.54. FD-MS: m/z = 966.16.

**1-(3-(Methoxycarbonyl)propyl)-1-(thieno[3,2-b]thiophen-2-yl)-[6,6]methanofullerene (TThCBM, 3c).** TThCBM (111 mg, 33%) was prepared by the same procedure as **3a**. ^1^H NMR (CDCl_3_): δ 7.68 (s, 1H, Ar), 7.49 (d, 1H, *J* = 5.3 Hz, Ar), 7.34 (d, 1H, *J* = 5.1 Hz, Ar), 3.68 (s, 3H, OCH_3_), 3.00 (t, 2H, *J* = 8.0 Hz, CCH_2_), 2.58 (t, 2H, *J* = 7.5 Hz, COCH_2_), 2.34-2.25 (m, 2H, CH_2_). ^13^C NMR (CDCl_3_): δ 173.41 (C=O), 148.00, 147.29, 145.65, 145.27, 145.19, 145.05, 144.86, 144.70, 144.66, 144.57, 144.24, 143.84, 143.81, 143.11, 143.05, 142.98, 142.96, 142.25, 142.17, 141.11, 141.02, 140.81, 139.42, 138.38, 138.24, 137.62, 127.86 (ArH), 124.52 (ArH), 119.68 (ArH), 79.65 (bridgehead C), 51.70 (OCH_3_), 46.56 (bridge C), 33.89 (CH_2_), 33.70 (CH_2_), 22.56 (CH_2_). IR (KBr): 2943, 1735 (C=O; s), 1459, 1429, 1343, 1249, 1187, 1173, 1080, 1059, 905, 826, 799, 755, 742, 700 cm^-1^. Anal. Calcd for C_72_H_12_O_2_S_2_: C, 88.88; H, 1.24; S, 6.59. Found: C, 87.49; H, 1.70. FD-MS: m/z = 972.50.

**1-(3-(Methoxycarbonyl)propyl)-1-(5-(ethylcarboxy)thieno[3,2-b]thiophen-2-yl)-[6,6]methanofullerene (es-TThCBM, 3d).** 214 mg, 42% yield. ^1^H NMR (CDCl_3_): δ 8.04 (s, 1H, Ar), 7.70 (s, 1H, Ar), 4.42 (q, 2H, *J* = 7.2 Hz, CH_3_C*H_2_*O), 3.69 (s, 3H, OCH_3_), 3.01 (t, 2H, *J* = 8.2 Hz, CCH_2_), 2.59 (t, 2H, *J* = 7.4 Hz, COCH_2_), 2.31-2.28 (m, 2H, CH_2_C*H_2_*CH_2_), 1.42 (t, 3H, *J* = 7.2 Hz, OCH_2_C*H_3_*). ^13^C NMR (CDCl_3_): δ 173.31 (C=O), 162.47 (C=O), 147.64, 146.95, 145.52, 145.27, 144.95, 144.85, 144.68, 144.61, 144.29, 143.81, 143.06, 143.01, 142.13, 141.06, 140.86, 138.71, 138.28, 138.24, 135.80, 125.69 (ArH), 124.77 (ArH), 79.28 (bridgehead C), 61.51 (OCH_2_), 51.73 (OCH_3_), 46.30 (bridge C), 33.79 (CH_2_), 33.63 (CH_2_), 22.54 (CH_2_), 14.40 (OCH_2_*C*H_3_). IR (KBr): 2942, 1736 (C=O; s), 1707 (C= ; s), 1491, 1429, 1365, 1341, 1276, 1238, 1167, 1069, 1017, 747 cm^-1^. Anal. Calcd for C_75_H_16_O_4_S_2_: C, 86.20; H, 1.54; S, 6.14. Found: C, 87.49; H, 1.70. FD-MS: m/z = 1044.03.

**References**

1. Fuller, L. S.; Iddon, B.; Smith, K. A. *J. Chem. Soc., Perkin Trans. 1* **1997,** 3465–3470. doi:[10.1039/a701877k](http://dx.doi.org/10.1039/a701877k)
2. Hummelen, J. C.; Knight, B. W.; LePeq, F.; Wudl, F.; Yao, J.; Wilkins, C. L. *J. Org. Chem.* **1995,** *60,* 532–538. doi:[10.1021/jo00108a012](http://dx.doi.org/10.1021/jo00108a012)
3. Popescu, L. M.; van’t Hof, P.; Sieval, A. B.; Jonkman, H. T.; Hummelen, J. C. *Appl. Phys. Lett.* **2006,** *89,* No. 213507. doi:[10.1063/1.2397003](http://dx.doi.org/10.1063/1.2397003)
